# A Behavioral Test of Accepting Benefits that Cost Others: Associations with Conduct Problems and Callous-Unemotionality

**DOI:** 10.1371/journal.pone.0036158

**Published:** 2012-04-27

**Authors:** Joseph T. Sakai, Manish S. Dalwani, Heather L. Gelhorn, Susan K. Mikulich-Gilbertson, Thomas J. Crowley

**Affiliations:** 1 Department of Psychiatry, University of Colorado Anschutz Medical Campus, Aurora, Colorado, United States of America; 2 United BioSource Corporation, Bethesda, Maryland, United States of America; University of Granada, Spain

## Abstract

**Background:**

Youth with conduct problems (CP) often make decisions which value self-interest over the interests of others. Self-benefiting behavior despite loss to others is especially common among youth with CP and callous-unemotional traits (CU). Such behavioral tendencies are generally measured using self- or observer-report. We are unaware of attempts to measure this tendency with a behavioral paradigm.

**Methods/Principal Findings:**

In our AlAn's (altruism-antisocial) game a computer program presents subjects with a series of offers in which they will receive money but a planned actual charity donation will be reduced; subjects decide to accept or reject each offer. We tested (1) whether adolescent patients with CP (n = 20) compared with adolescent controls (n = 19) differed on AlAn's game outcomes, (2) whether youths with CP and CU differed significantly from controls without CP or CU, and (3) whether AlAn's game outcomes correlated significantly with CP and separately, CU severity. Patients with CP and CU compared with controls without these problems took significantly more money for themselves and left significantly less money in the charity donation; AlAn's game outcomes were significantly correlated with CU, but not CP.

**Conclusions/Significance:**

In the AlAn's game adolescents with conduct problems and CU traits, compared with controls without CP/CU, are disposed to benefit themselves while costing others even in a novel situation, devoid of peer influences, where anonymity is assured, reciprocity or retribution are impossible, intoxication is absent and when the “other” to be harmed is considered beneficent. AlAn's game outcomes are associated with measures of CU. Results suggest that the AlAn's game provides an objective means of capturing information about CU traits. The AlAn's game, which was designed for future use in the MRI environment, may be used in studies attempting to identify the neural correlates of self-benefiting decision-making.

## Introduction

Youth with conduct problems (CP) often make decisions which appear to value self-interest over the interests of others. For example, several criteria for the clinical diagnosis of conduct disorder involve taking at the expense of others: i.e., stealing with or without confrontation, forced sex, and lying for goods or favors or to avoid obligations. Such self-benefiting behavioral tendencies are generally measured by self- or observer-report. Although we are unaware of behavioral paradigms which seek to measure this tendency toward self-benefiting actions in youth with conduct problems, some behavioral measures of altruism require subjects to weigh the interests of others against self-interest [1]; for example, Harbaugh et al. [2] asked subjects to decide whether to donate to a charity (a local food bank), despite a cost to themselves; some trials were voluntary, while others mandated donations, akin to taxation. Moll et al. [3] examined decisions to donate, or oppose donation, to a series of charities. Some decisions were costly-to-subjects and others were non-costly. These games, although of great interest, did not measure taking for self at the expense of others. The Dictator Game requires one subject to decide on a split of money between the “dictator” and another player [4], [5]. Thus subjects may make more self-benefiting or other-benefiting decisions. Here, building from that important work on altruism, we sought to develop a game which could be used in future fMRI studies and where subjects repeatedly decided whether or not to pursue actions that result in self-benefit while costing others.

Any behavioral measure of tendency toward antisocial behavior problems must contend with the following: (1) youths may develop the same antisocial phenotype (such as conduct problems (CP)) through various etiologies; and, (2) youths with CP represent a heterogeneous group [6], [7]. This heterogeneity may be reduced by focusing on CP youths with callous and unemotional (CU) traits, as these traits apparently identify an important subgroup of antisocial youths [7]. It's important to note that CU and CP are associated, but distinct traits. Many youths with CP have CU but some do not. Similarly, many youths with CU have CP but some do not.

Among youths with CP, CU is associated with expectation of more instrumental gain and positive consequences from aggressive actions together with decreased expectancies of negative consequences of deviant behavior [8], [9]. CU traits predict more severe CP and aggression, persistence of symptoms [6], future violent behavior and poorer treatment outcomes [10]. Many longitudinal studies now demonstrate relatively high stability of CU ratings throughout childhood, from childhood into adolescence, and from late adolescence into young adulthood [11]. Some work also supports that individuals with CP and CU traits demonstrate less emotional empathy or positive affect, [8], and exhibit deficits in emotional processing [12] and moral reasoning [13]. Because of these emotional deficits, it is hypothesized that such youth, throughout development, are less able to learn from others' responses to their actions, thereby impacting conscience development [14]. Perhaps because of this poor emotional responsivity, these individuals are less likely to constrain behaviors which harm others [13]. CP with CU is highly heritable, while CP alone is only moderately heritable [15]. Thus, among youth with CP, CU traits appear to identify a particularly severe and persistent subgroup of individuals who may possess a more biologically-based and less environmentally-influenced trait. Thus, self-benefiting behavior despite cost to others may be especially common among CP youth with CU. Such work underscores the importance and potential value of measuring CU traits in studies of adolescent antisocial phenotypes and considering youth with CP and CU separately from youth with CP without CU in some analyses.

Decisions to choose self-benefiting behaviors despite harm to others may be due to a number of situational or dispositional factors. These include social reinforcement and peer influences [16], situational anonymity or lack of concern for reputation [17]–[19], perceived likelihood of retribution or reciprocal altruism [20], [21], substance intoxication [22], characteristics of the “other” to be helped or harmed [23], [24], and opportunity. We designed and tested a new paradigm, the AlAn's (altruism-antisocial) game, in which subjects accept or reject a series of offers that include both benefit to self and cost to others, for determining whether youths with CP are disposed toward self-benefiting behaviors that harm others, even in a novel situation, without peer influences, where anonymity is assured, reciprocity or retribution are impossible, intoxication is absent, when the “other” to be harmed is considered beneficent, and opportunity is equal across all individuals. We tested (1) whether a disposition to benefit oneself at a cost to others differs between adolescent patients with CP and adolescent controls (between-group analyses), and (2) whether behavior on the AlAn's game correlates with CP severity (dimensional analyses). Because our paradigm requires decision-making where subjects weigh self-interest against other's interests, and because of the extant literature underscoring that youth with CP and CU traits demonstrate less emotional empathy, we further tested (3) whether youths with CP and CU differ significantly from controls without CP or CU (between-group analyses), and (4) whether behavior on the AlAn's game correlates with CU trait scores. In very early game development, debriefing showed that many individuals (non-clinical) deemed as appropriate accepting trials in which benefit to the player exceeded loss to a charity. Therefore, we also hypothesized that CP patients would especially differ from controls in trials that involved large losses to a charity and small rewards to the player.

## Materials and Methods

### Participants

After Colorado Multiple IRB approval, twenty adolescent patients (15 male) were recruited through a university-based adolescent treatment program. Patients are generally referred to treatment through social services or criminal justice agencies. Previous publications support that most of the patients admitted to this program have both conduct disorder and substance dependence by DSM-IV criteria [25]; treatment focuses on both these disorders, utilizing behavioral approaches, family-based therapy and medications for comorbid disorders such as depression. Nineteen control adolescents (15 male) were recruited through flyers, advertisements in school newspapers and a market research company. Written informed consent (parents) and assent (adolescents) was obtained from all research subjects. Inclusion criteria for both groups were: 1) age 14–18 years, 2) primary language is English, 3) reading proficiency adequate to understand consent, 4) not mentally retarded, 5) not threatening or dangerous, 6) no physical illness that would exclude participation, 7) adolescent and all first degree relatives have never worked, or volunteered for, or received assistance from the Red Cross, and 8) parent or guardian has adequate English proficiency to provide informed consent and complete the written questionnaires. Recruitment of control subjects was monitored to assure controls had similar age, sex, racial and ethnic distributions to patients and that controls lived in a zip code from which patients are usually referred. Exclusion criteria for controls were: 1) history of court conviction for offenses other than minor traffic or curfew violations, and 2) history on pre-evaluation screening of substance-related treatment or substance-related expulsion from school.

Table 1 shows that patients and controls (Section 1) and separately patients-with-CU and controls-without-CU (Section 2) did not significantly differ in terms of age, sex, race, ethnicity or socioeconomic status. The average age for both groups (patients and controls) was about 16 years, about three-quarters were male, two-thirds Caucasian, and about 15% Hispanic.

**Table 1:** Between group comparisons – demographics.

			Section 1:			Section2:		
			Pts (n = 20)	Cts (n = 19)	p-value	Pts-CU (n = 14)	Cts-NoCU (n = 16)	p-value
**Demographics:**	Age (years)		15.9 (0.99)	15.7 (0.75)	0.56	15.9 (0.83)	15.8 (0.77)	0.55
	Sex: male		75.0% (15)	78.9% (15)	>0.99^a^	78.6% (11)	75% (12)	>0.99^a^
	Race	White	65.0% (13)	68.4% (13)	0.82^b^	64.3% (9)	68.8% (11)	0.80^b^
		African American	15.0% (3)	15.8% (3)		7.1% (1)	12.5% (2)	
		Other/mixed race	20.0% (4)	15.8% (3)		28.6% (4)	18.8% (3)	
	Hispanic		15% (3)	16% (3)	>0.99^a^	14.3% (2)	18.8% (3)	>0.99^a^
	SES		41.7 (12.23)	35.3 (11.39)	0.10	41.6 (12.83)	36.6 (12.02)	0.29

Section 1- all patients (n = 20) and all controls (n = 19); Section 2 – subset of patients with callous unemotional traits (CU) and controls without CU; mean (sd) or % (n).

**Abbreviations:** Cts = controls; CU = utilizing questions 3,5,6 and 8 from the ICU we determined whether subjects would qualify for the Callous and Unemotional Specifier for Conduct Disorder recently proposed for inclusion in the Diagnostic Statistical Manual-V (DSM-V); NoCU = not meeting the Callous and Unemotional Specifier; Pts = patient; SES = socioeconomic status.

**Footnotes:**

a Fisher's Exact Test;

b due to small cell sizes, comparison is of whites vs. all others.

### Measures

Measures in the study assessment battery included:

1. A measure of socioeconomic status (SES) modified from Hollingshead & Redlich [26]: this measure has been used in our previous studies [25] and utilizes parent-reports of the primary wage earner's education and occupation; both of these variables are assigned a rank of 1–7 according to categories, and then ranks are weighted and summed according to procedures previously described, yielding a range of scores from 11–77, with 11 representing the highest SES [26].
2. The Child Behavior Checklist (CBCL) [27]: this parent-report assessment is standardized by gender and for ages 4–18 and provides dimensional ratings of conduct problems in children. CBCL was included as one of our two measures of severity of conduct problems.
3. The Youth Self Report (YSR) [28]: this self-report assessment produces dimensional ratings of conduct, attention, and affective problems, with excellent reliability and validity, and provides dimensional ratings on several problem scales that are standardized by gender and age. The YSR has shown discriminative validity for conduct problems [29] and was included, along with CBCL, as a measure of severity of conduct problems.
4. The Inventory of Callous and Unemotional Traits (ICU) [30]: the ICU has been validated in adolescent offenders [31] and in large non-referred samples [32]; many longitudinal studies now support the reliability of ICU ratings throughout childhood and from childhood into adolescence [11]. This self-report instrument provided one of our two measures of callous-unemotionality (range 0–72).
5. The Antisocial Process Screening Device (APSD) [33]: this 20-item self-report instrument, based on the Psychopathy Checklist-Revised [34], identifies more severe and chronic juvenile offenders [35]. Confirmatory factor analyses support a 3-factor structure, including dimensions of narcissism, impulsiveness and callous-unemotionality [36]; this last subscale provided our second measure of callous-unemotionality (CU subscale range 0–12).
6. The Social Desirability Scale (SDS) [37]: this instrument was included to assess whether groups varied in their tendency to provide apparently socially desirable, rather than internally motivated, responses.

### Procedures

A study research assistant met with the adolescent subject and parent during Meeting 1. Following informed consent, parents completed the CBCL [27], a race/ethnicity questionnaire, and a measure of socioeconomic status [25], [26]; adolescent subjects completed the YSR [28], provided a urine sample for onsite drug testing (iCup®, Instant Technologies, Inc., Norfolk, VA) and a breath test for alcohol (Alco-Sensor III™, Intoximeters Inc., St. Louis, MO).

Approximately 3–7 days later, eligible subjects completed Meeting 2 in a quiet private room, chosen by the subject, in the subject's home, a public facility (i.e. library), or our research offices. Subjects again provided urine and breath samples for onsite drug and alcohol testing. Then they viewed an investigator-produced ninety second slide presentation with pre-recorded messages highlighting the positive contributions of the Red Cross. After viewing the slides, subjects indicated how much good they felt the Red Cross does by marking on a line anchored by the terms “No good at all” and “Lots of good” (Red Cross Visual Analogue Scale). Subsequently, subjects viewed the AlAn's game instructions (slides with pre-recorded audio; instructions are included in Material S1), and then answered questions about the game to ensure comprehension of the instructions. All participants played a short practice version of the game to familiarize them with the timing and format of the game. We told all participants that we would hold research data in strict confidence. Subjects had the opportunity to ask questions or to replay the practice game. Then subjects played the AlAn's game, which takes approximately 31 minutes. By protocol, seating arrangements ensured that the laptop screen was only visible to the subject. After finishing the game, subjects completed a short debriefing interview and then several self-report measures including: the Inventory of Callous-Unemotional Traits (self-report) [30]; the Antisocial Process Screening Device (self-report) [33]; and the Social Desirability Scale [37]. Qualitative data from our debriefing interviews supported that subjects were generally engaged by and understood the AlAn's game. Four research assistants administered the study protocol; each conducted a similar proportion of patient and control interviews.

### Description of the AlAn's (Altruism/Antisocial) Game

This game shares some similarities with previous tests of altruism [1]–[3] or behavioral economic games [4], [5]. However, the AlAn's game differs from such games in several fundamental ways. First, in altruistic acts the actor *gives* up something *to benefit others* [38]. In contrast, many antisocial acts *take* something from others *to benefit the actor*; therefore, in the AlAn's game we offered subjects the opportunity to take some benefit despite expense to another. Second the game was designed to be quantitative, allowing between group comparisons of outcomes. Subjects started with no money; a planned, real donation to the Red Cross started at $16 but could decrease due to choices made by the participant playing the game. Subjects were told that the researchers would (and they did) give the Red Cross the value of the charity donation remaining at the end of the game. Based on previous studies which have demonstrated differences between hypothetical and actual valuations [39]–[41], we designed AlAn's game to use actual money donations to a real charity. Third, we designed the game for future use in the fMRI environment; for example, we included many (72) decision-making trials and also trials which may be used as a contrast in MRI designs (Calculation Trials). Finally, we utilized a single charity, which we hypothesized would generally be considered beneficent; this single “other” standardized the experience across subjects.

We carefully considered whether to utilize a second participant, or a real person who was not a research participant, instead of a charity; empathy and perspective-taking generally are conceptualized as a between-individual, and not person-charity, phenomena. However, we opted to use a charity for these reasons: (1) given that empathy can vary based on how similar the “other” is to one's self (i.e. ingroup vs. outgroup), we worried that failure to appropriately match the “other” to the research participant would introduce error (i.e. subject made more self-benefiting choices because he considered the other player to be very dissimilar to him, rather than because of an inherent tendency toward antisocial behaviors); and, (2) some of our adolescent patients may use manipulation or threats of aggression in interpersonal interactions, or may address grievances through violence. Thus, we worried about pairing adolescent patients together or with controls while playing a game requiring choices that pit self interest against the interests of others. Although paradigms have been constructed where two participants remain anonymous to one another, and even to the researchers [4], we wanted research participants to view the “other” as beneficent and deserving, given that increasing the perceived “deservingness” of the other in economic games increases the quantity of donations from experimental subjects [5]. Thus, we chose to utilize a single charity that we thought would be broadly viewed as beneficent, to standardize the experience across subjects.

Subjects were told directly that the researchers were interested in understanding how people make decisions when they can win but others will lose. No attempts were made to deceive subjects and no cover story was utilized. Instead subjects were informed that there were no “right” or “wrong” answers while playing the game and that all information about how they played the game would be held in strict confidence; we would not inform parents about how subjects played the game, and for patients we would not inform treatment providers.

**Figure 1:**
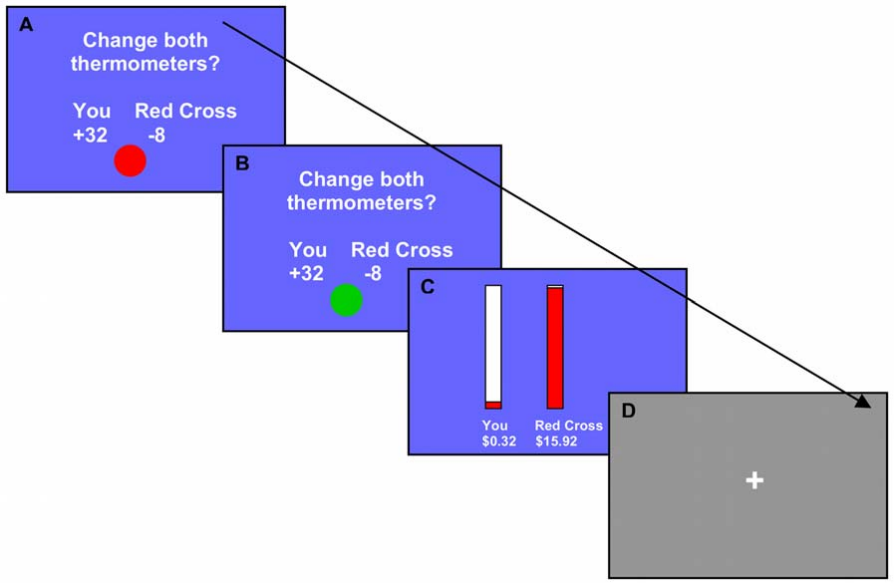
AlAn's game trials: screen-captures with associated timing (example Active Trial shown). Note: **A**. An offer in which the subject will receive 32 cents and the Red Cross donation will be reduced by 8 cents (5 seconds); **B**. When the red ball turns green, subjects have one second to press one computer key to accept the offer, or a different key to reject it; **C**. The thermometers and counters then show how much money the subject has accrued. Here the subject accepted so the “You” counter now reads 32 cents and the “Red Cross” counter reads $15.92); **D**. Gray fixation screen.

Subjects made 144 choices (“trials”) divided into two 72-trial sessions. Each trial was approximately 13 seconds in length (see Figure 1): 5 seconds to view and consider the offer, 1 second to press an “accept” or “reject” computer key, 4 seconds to observe results, and 2–4 seconds of a gray fixation screen. The “results” portion of each trial showed the outcomes of the choice; analogue “thermometers” and digital counters showed the subject's accrued money and the current remaining value of the Red Cross donation.

**Figure 2:**
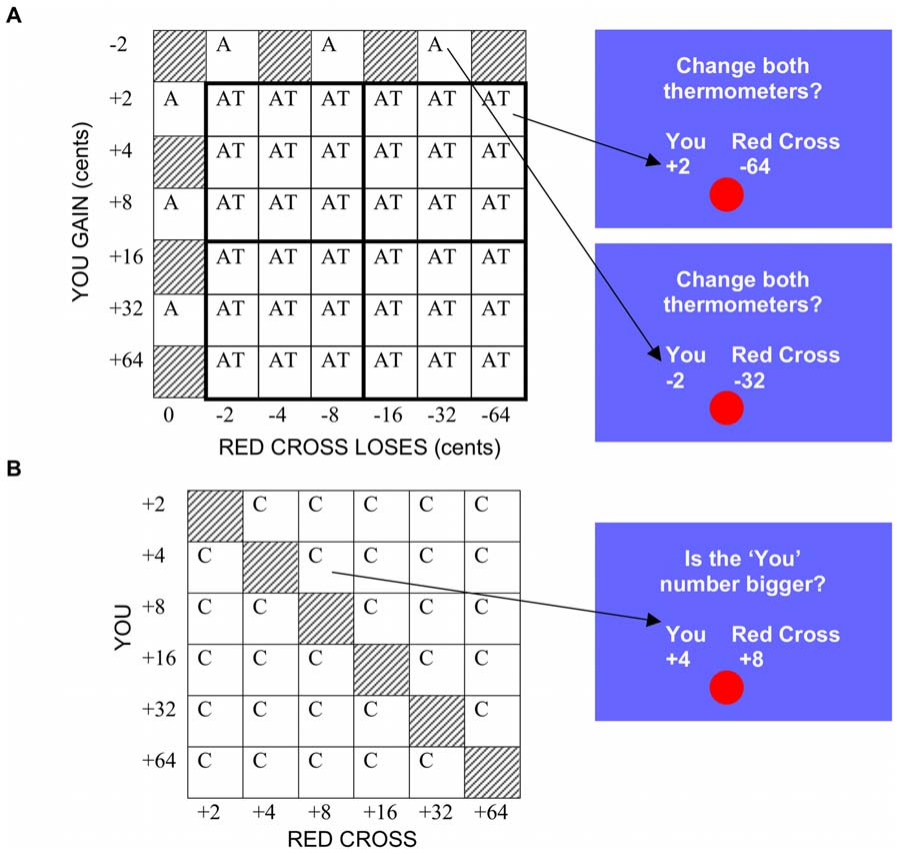
The AlAn's game: description of three trial types. **2.A**: Matrix with “You Gain” values (Y axis) and “Red Cross Loses” values (X axis) in cents. Each cell in the matrix represents a single offer. **AT** = active trials; arrow from AT points to a screencapture showing a sample active trial in which “You” will get 2 cents but the Red Cross donation will decrease by 64 cents. Note that the “You” amount varies independently from the “Red Cross” amount. **A** = Attention-Control; trials in which either a “Yes” results in a loss for both subject and Red Cross (top row of matrix), or a “Yes” results in gain for the subject and no loss for the Red Cross (first column of matrix); arrow from A points to a screencapture showing a sample Attention-Control trial in which “You” will lose 2 cents and the Red Cross donation will decrease by 32 cents. Note: Thicker borders indicate the quadrants for which percentage of “Yes” responses across those 9 Active Trials were calculated (see Materials and Methods, Data Analyses, Between-Group analyses). **2.B: C** = Calculations, in which subjects decide whether the “You” number is bigger than the Red Cross number; arrow points to a screencapture showing a sample calculation trial in which the “You” number (4 cents) is not bigger than the “Red Cross” number (8 cents).

The AlAn's game contained three trial types (See Figure 2). During Active Trials (“AT” in Figure 2.A) the decision screen showed an amount that the subject would gain (range 2–64 cents) and the Red Cross would lose (same range) if the choice were accepted (“Change both thermometers?”); in most trials the two amounts were unequal. Subjects accepted (“Yes” button) or rejected (“No” button) the choice on the screen; after rejected choices neither counter changed. Attention-Control Trials (“A” in Figure 2.A) have a correct or logical answer. We assumed that most subjects would reject all trials in which both they and the Red Cross would lose money, so these trials forced changes if a subject fell into a repetitive pattern of accepting all offers (to maximize earnings). Conversely, we assumed most subjects would accept all choices in which they would get money and the Red Cross donation would not be reduced, so these trials forced changes if a subject fell into a repetitive pattern of rejecting all offers (to prevent any loss to the Red Cross). These trials also objectively tested subject attention to the task. Calculation Trials (“C” in Figure 2.B) ask, “Is the YOU number bigger?” while presenting one positive number for the subject and another for the Red Cross. Subjects were instructed to press the YES button if the subject's number (the “YOU” number) was bigger than the Red Cross' number, and the NO button if the subject's number (the “YOU” number) was not bigger than the Red Cross' number. These trials assessed whether patients could, in 6 seconds, understand relative numerical values used in the Active Trials. In addition, the AlAn's game was designed for future use in the MRI environment which further motivated inclusion of multiple Calculation Trials. Calculation Trials, compared with Active Trials, present the same visual cues, require the same motor responses, and require subjects to judge the relative numerical values but are devoid of self vs. other consideration. Thus they may serve as a comparison for Active Trials in future MRI studies. Each of the two sessions contained 36 Active Trials, 6 Attention-Control Trials and 30 Calculation Trials (72 per session, 144 total). The order of amounts offered (order of trial presentation) was randomized before the beginning of the study and was the same for all subjects.

Subjects received $15 for their time and effort. To provide real monetary consequences to their within-game decisions, they were given their earnings from the game (Active and Attention-Control Trials). Money left to the Red Cross in the game actually was donated to the Red Cross. To incentivize attention to Calculation Trials, subjects received $0.25 if they correctly answered at least 75% of the Calculation Trials. Subjects were paid in cash at the end of Meeting 2.

### Data Analyses

The AlAn's game, programmed in EPRIME (EPRIME V2.0, http://www.eprime2.eu/), provided output files with all variable names denoting trial type and choices made by each participant. We double entered self-report measures into a Microsoft Access database, imported both AlAn's game and self-report data into SPSS [42], and checked for inconsistencies.

#### Between-group analyses (patients with CP vs. controls without CP)

We compared patients and controls for demographics (age, sex, race, ethnicity, socioeconomic status) to ensure between-group similarity. We also compared groups for the Inventory of Callous and Unemotional Traits (ICU), Antisocial Process Screening Device (APSD), Red Cross Visual Analogue Scale, Social Desirability Scale, and CBCL/YSR DSM-oriented conduct problems T-scores, utilizing independent t-tests and chi square tests or their nonparametric equivalents (Mann-Whitney U and Fisher's Exact tests, respectively) when variables violated normality assumptions.

To test whether the AlAn's game could discriminate between patients and controls several strategies were employed. First, using Mann-Whitney U tests we compared patients and controls for differences in AlAn's game outcomes: “You”, the amount of money taken for oneself while playing the game and “RC”, the amount of money left in the charity donation at the end of the game. Second, to visually examine within-group behavior by trial characteristics (i.e. for each cell in Figure 2.A), we calculated the percent of trials accepted for each combination offered in Active Trials (e.g. You get 2 cents and Red Cross loses 64 cents) within patients and separately, within controls; the game presented each choice to each subject twice. Next, we calculated patient-control group differences in percent of trials accepted for each Active Trial. We hypothesized that patients would accept significantly more relatively-costly-to-Red-Cross trials (i.e. large Red Cross subtractions and small rewards to self) than would controls. Trials on the right side (Figure 2.A, columns 5–7; e.g. You Get 8 cents, Red Cross donation decreases by 64 cents) are relatively more costly to Red Cross, and those on the left side (Figure 2.A, columns 2–4; e.g. You Get 8 cents, Red Cross donation decreases by 2 cents, Figure 2.A) are relatively less costly to Red Cross. To evaluate statistically the relationships between Red Cross loss and self (“You”) gain, we divided Active Trials into two levels by magnitude of loss to Red Cross (“Low” -2 to -8; “High” -16 to -32) and into two levels by magnitude of gain to “You” (“Low” 2 to 8; “High” 16 to 32), creating a “Red Cross loss” and “You” gain effect, respectively, and creating four quadrants of active trials: upper left (small “You” gain, small Red Cross (RC) loss), upper right (small “You” gain, large RC loss), lower right (large “You” gain, large RC loss), and lower left (large “You” gain, small RC loss). For each subject, we calculated percentage of “Yes” answers for the 9 active trials comprising each quadrant and evaluated them as repeated measures in a mixed model ANOVA. Fixed effects included a between subject effect of group (patient, control) and within-subject effects of Red Cross loss (low, high) and “You” gain (low, high) with a random effect of subject nested within group. The repeated measures on subject (averaged within quadrant) were assumed to have an unstructured covariance structure. Interactions among effects were evaluated with a manual step-down (backward regression) procedure where the most non-significant higher-order term was removed and the model was re-run, beginning with removal of the highest-order three-way interaction term: group*Red Cross loss*“You” gain. Likelihood ratio tests and minimum values for Akaike's Information Criterion (AIC) determined the best model [43].

#### Between-group analyses (patients with CP and CU vs. controls without CP or CU)

Because CU traits identify a subgroup of youth with CP with severe and persistent antisocial behavior [11], investigators commonly divide CP and control youth into those with and without CU traits [6], [44]–[45]. Although some investigators use a median split or other techniques for dividing samples based on CU severity, we chose the following approach: we utilized questions 3, 5, 6 and 8 from the ICU and recoded responses on a 0–3 scale with 3 indicating greater callous-unemotionality; items 3, 5 and 8 are positively worded items, requiring reverse scoring. For each of the 4 items, a score of 2 or 3 was sufficient to meet that criterion and subjects had to meet at least 2 of the 4 criteria to meet the callous-unemotional specifier. Although other approaches have been utilized, we are only aware of one standard method for identifying CU traits which was recently proposed for the DSM-V [46]. We followed those proposed procedures here because (1) we are unaware of other standardized methods of categorization of CU traits and (2) given that this approach may be incorporated into DSM-V, in the future it is likely to be broadly utilized by clinicians and researchers. Most patients (n = 14/20) met requirements for the CU specifier and few controls did (n = 3/19). Between-group analyses described above (paragraphs 2 and 3 of this section) were also completed to compare patients with CU traits (n = 14) and controls without CU traits (n = 16).

#### Dimensional analyses

To assess the relationship between AlAn's game outcomes and CP and CU measures, we computed Spearman's rank-order correlations between total AlAn's game earnings (“You”), the ending Red Cross donation (RC), YSR and CBCL DSM-oriented conduct problems T-scores, Antisocial Process Screening Device (APSD) scores, and scores on the Inventory of Callous and Unemotional Traits (ICU). Correlations were also completed for other APSD and ICU subscales and although not presented, are available to interested readers upon request. Regression analyses were employed to investigate the relationship between ICU total score (dependent variable) and AlAn's game (independent variable; percent of “Yes” answers to Active Trials). The initial model included ICU total score, AlAn's game and group (patient vs. control). Additional covariates (internalizing and externalizing problem scores from the CBCL, age, sex, socioeconomic status, race (white vs. other) and ethnicity (Hispanic yes vs. no) were added to see if covariates explained the observed relationship between ICU and AlAn's game.

***AlAn's game performance:*** Finally, to test for group differences in the ability to understand and play the AlAn's game, we evaluated group differences in performance on Attention-Control Trials (attention to the task) and Calculation Trials (ability to determine relative numerical values).

## Results

### Between-Group: Comparing Patients with CP vs. Controls without CP

Table 2 (section 1, columns 4–6) shows that patients scored significantly higher than controls on measures of callous-unemotional traits. Patients and controls did not differ on their Red Cross Visual Analog Scale, in which subjects were asked to rate “How much good do you think the Red Cross really does?”; each group averaged about 85 (100 indicating “Lots of good”) confirming that the Red Cross was viewed positively in both groups. As expected, patients scored significantly higher than controls on parent-rated and self-report measures of conduct problems (both p<0.001).

**Table 2:** Between group comparisons: callousness and AlAn's game.

			Section 1:			Section2:		
			Pts (n = 20)	Cts (n = 19)	p-value	Pts-CU (n = 14)	Cts-NoCU (n = 16)	p-value
**CU-measures**	ICU		30.5 (9.68)	23.4 (7.10)	0.01*	33.7 (9.63)	21.5 (4.98)	<0.001*
	APSD	Callous	4.9 (1.31)	3.2 (1.44)	0.001*	5.1 (1.41)	2.8 (1.05)	<0.001*
**Other measures:**	Red Cross VAS^a^		84.6 (17.52)	85.4 (11.63)	0.74^b^	88.1 (10.52)	86.4 (10.61)	0.64^b^
	Social Desirability Scale		15.0 (5.56)	15.8 (5.22)^c^	0.58	14.4 (5.43)	15.9 (5.42)^c^	0.45
	CP_CBCL_		77.0 (8.41)	53.3 (4.19)	<0.001*	77.4 (8.77)	53.6 (4.46)	<0.001*
	CP_YSR_		69.8 (7.29)	55.2 (5.18)	<0.001*	71.4 (5.76)	55.4 (5.25)	<0.001*
**AlAn's game Outcomes:**	You		$13.9 (2.45)	$12.8 (3.14)	0.32^b^	$14.1 (2.73)	$12.2 (3.06)	0.04^b^ *
	RC		$6.4 (4.62)	$8.7 (5.28)	0.19^b^	$5.4 (4.43)	$10.2 (4.35)	0.01^b^ *

Section 1- all patients (n = 20) and all controls (n = 19); Section 2 – subset of patients with callous unemotional traits (CU) and controls without CU; (mean (sd);

* p<0.05);

**Abbreviations:** APSD = Antisocial Process Screening Device; CP_CBCL_ = Diagnostic and Statistical Manual-oriented conduct problems scale T-score from the CBCL; CP_YSR_ = Diagnostic and Statistical Manual-oriented conduct problems scale T-score from the Youth Self Report; Cts = controls; CU = utilizing questions 3,5,6 and 8 from the ICU we determined whether subjects would qualify for the Callous and Unemotional Specifier for Conduct Disorder recently proposed for inclusion in the Diagnostic Statistical Manual-V (DSM-V); ICU = Inventory of Callous and Unemotional Traits; NoCU = not meeting the Callous and Unemotional Specifier; Pts = patient; RC = ending Red Cross donation; VAS = Visual Analogue Scale; You = money taken for self while playing the AlAn's game;

**Footnotes:**

a Subjects are asked “How much good does the Red Cross do?”, scale is measured from 0–100 with 0 = “No good at all” and 100 = “Lots of good”;

b Mann-Whitney U test;

c 2 control subjects did not complete all questions on the SDS and were not included in these analyses.

**Figure 3:**
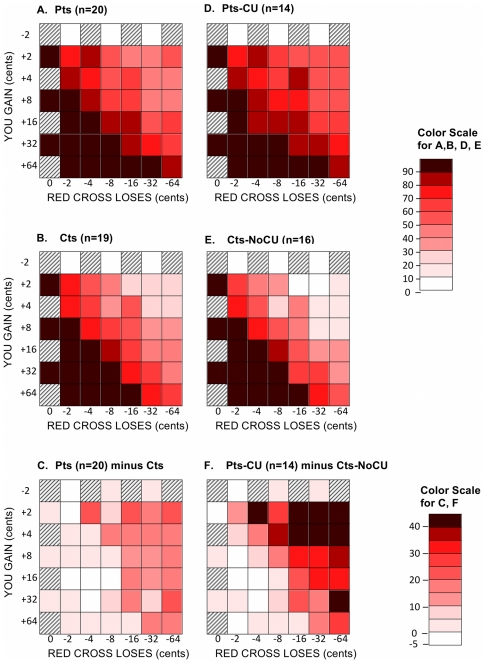
“Heat” maps showing percentage of “Yes” Answers in A, B, D and E. Figure 3.C, F, Group differences in acceptance of AlAn's Game offers. Legend: Matrix with “You Gain” values (Y axis) and “Red Cross Loses” values (X axis) in cents. Each cell in the matrix represents a single offer. The first column and top row of the matrix are Attention-Control Trials (see Methods). For each cell, each subject's percent of “Yes” responses was calculated across the 2 sessions. Those values were entered into a grand mean for each group (i.e. Patients and separately, Controls) for each cell. Exact percentages are shown in Figure S1, while percentages here are represented by a color scale. Each cell of Figure 3.C presents the patient-minus-control difference of the corresponding cells of Figure 3.A and 3.B. Each cell of Figure 3.F presents the patient-CU-minus-control-NoCU difference of the corresponding cells of Figure 3.D and 3.E. Pts = patients; Cts = controls; CU = utilizing questions 3,5,6 and 8 from the ICU we determined whether subjects would qualify for the Callous and Unemotional Specifier for Conduct Disorder recently proposed for inclusion in the Diagnostic Statistical Manual-V (DSM-V); NoCU = not meeting the Callous and Unemotional Specifier.

Table 2, Section 1, also shows that patients and controls did not differ significantly on overall AlAn's game outcomes (“You” or amount of money taken for self while playing the game; p = 0.32; and, “RC” or the amount of money left in the charity donation at the end of the game; p = 0.19). Figure 3 presents a “heat” map of percent of accepted offers by trial characteristics within group (patients 3.A and controls 3.B – note actual percent values are provided in Figure S1). Figure 3.C shows the difference for each cell (patient group minus control group; 3.A–3.B). Our hypothesis was that patients, compared with controls, would make more choices which resulted in relatively low self-benefit and large cost to others. That general pattern of patient-control difference can be seen visually in Figure 3.C. We divided Active Trials into four quadrants: upper left (small “You” gain, small Red Cross (RC) loss), upper right (small “You” gain, large RC loss), lower right (large “You” gain, large RC loss), and lower left (large “You” gain and small RC loss) and calculated percentage of “Yes” answers for each quadrant for each subject. Mixed model analysis of variance (ANOVA) evaluated statistically the relationships between Red Cross loss (low, high) and “You” gain (low, high) on percentage of “Yes” answers in each quadrant. The final model included group (F_1,39_ = 1.71, p = 0.1991), Red Cross loss (F _1,117_ = 72.00, p<.0001), “You” gain (F _1,117_ = 85.50, p<.0001), and group*Red Cross loss (F _1,117_ = 3.49, p = .0641). From this model, subjects averaged 84.0 percent “Yes” responses to active trials with high “You” gain compared to 55.9 percent “Yes” responses to active trials with low “You” gain. Controls and patients both averaged greater than 80 percent “Yes” responses when Red Cross loss was low (80.6, 85.1, respectively); when Red Cross loss was high, controls averaged 49.1 and patients averaged 64.9 percent “Yes” responses to active trials. While “You” gain and Red Cross loss values were significant in the model, groups did not differ significantly in their behavior.

### Between Group: Comparing Patients with CP and CU vs. Controls without CP or CU

Following the example of others [6], [44], [45], we divided groups based on severity of CU; we selected patients with, and controls without, the CU specifier proposed for inclusion in the DSM-V. We then repeated analyses described above (in the previous paragraph). Table 2 (section 2, columns 7–9) shows patients with CU traits (n = 14) and controls without CU traits (n = 16) were similar for Red Cross Visual Analog Scale, and the Social Desirability Scale scores. On the AlAn's game, patients with CU (Table 2, section 2) took significantly more money (p = 0.04) and left significantly less money in the charity donation (p = 0.01) when compared with controls without CU. Figure 3.D–E shows acceptance rates by trial characteristics for patients with CU (3.D) and controls without CU (3.E); Figure 3.F shows patients with CU minus controls without CU (i.e. 3.D minus 3.E). Both groups accepted similar numbers of offers with high self-benefit and relatively little cost to others (small reductions from the charity donation); but patients with CU accepted more offers than controls without CU (Figure 3.F) when self-benefit was small and deductions from the charity donation were large. For example, patients with CU accepted about 57% of trials in which they would get 2 cents and the Red Cross donation would be reduced by 64 cents; controls without CU accepted about 13% of those trials (see Figures 3.D and 3.E; also see Figure S1). Finally, for patients with CU traits vs. controls without CU traits, a mixed model ANOVA (n = 30) evaluated statistically the relationships between Red Cross loss (low, high) and “You” gain (low, high) on percentage of “Yes” answers. The final model included group (F_1,30_ = 6.86, p = 0.0137), Red Cross loss (F _1,90_ = 71.12, p<.0001), “You” gain (F _1,90_ = 75.34, p<.0001), group*Red Cross loss (F _1,90_ = 11.05, p = .0013) and group*“You” gain (F _1,90_ = 8.11, p = .0055). When Red Cross loss was low, controls averaged 77.0 and patients averaged 88.9 percent “Yes” responses; when Red Cross loss was high, controls averaged 40.0 and patients averaged 72.0 percent “Yes” responses. When “You” gain was low, controls averaged 40.0 and patients averaged 70.6 percent “Yes” responses; when “You” gain was high, controls averaged 76.6 and patients averaged 89.1 percent “Yes” responses. Thus, as both “You” gain increased (low to high) and Red Cross loss decreased (high to low) subjects were significantly more likely to accept trials. Patients with CU traits, compared with controls without CU traits, significantly differed in their behavior (main effect); group differences become more apparent in the upper right quadrant where “You” gain is low and Red Cross loss is high (interactions are significant in the model).

### Dimensional: Associations of AlAn's Game with CP, and separately CU

Table 3 presents correlations of the amount of money left in the charity donation (RC) after playing the AlAn's game with the YSR DSM-oriented conduct problems T-scores and the CBCL DSM-oriented conduct problems T-scores. Those correlations were non-significant. Results for “You” (i.e. AlAn's game earnings) were similarly uncorrelated with these CP measures.

**Table 3:** Spearman's rank-order correlations of AlAn's game outcomes with callous and unemotional traits, and conduct problems measures.

BOTH GROUPS (n = 39)						
	You	RC	APSD_Call_	ICU_Tot_	CP_CBCL_	CP_YSR_
You	1	−0.95**	0.44**	0.44**	0.06	0.10
RC		1	−0.51**	−0.54**	−0.14	−0.16
APSD_Call_			1	0.76**	0.54**	0.47**
ICU_Tot_				1	0.32*	0.51**
CP_CBCL_					1	0.64**

WITHIN PATIENTS (n = 20)						
	You	RC	APSD_Call_	ICU_Tot_	CP_CBCL_	CP_YSR_
You	1	−0.90**	0.35	0.29	−0.07	0.001
RC		1	−0.43	−0.40	0.06	0.01
APSD_Call_			1	0.75**	0.06	0.18
ICU_Tot_				1	−0.02	0.42
CP_CBCL_					1	−0.27

WITHIN CONTROLS (n = 19)						
	You	RC	APSD_Call_	ICU_Tot_	CP_CBCL_	CP_YSR_
You	1	−0.96**	0.48*	0.41	−0.16	−0.24
RC		1	−0.51*	−0.51*	0.12	0.17
APSD_Call_			1	0.68**	0.12	0.07
ICU_Tot_				1	0.18	0.36
CP_CBCL_					1	0.24

* 0.01<p<0.05;

** p<0.01.

**Abbreviations:** APSD = Antisocial Process Screening Device; Call = Callousness subscale; CP_CBCL_ = Diagnostic and Statistical Manual-oriented conduct problems scale T-score from the CBCL; CP_YSR_ = Diagnostic and Statistical Manual-oriented conduct problems scale T-score from the Youth Self Report; ICU = Inventory of Callous and Unemotional Traits; RC = remaining balance in the Red Cross donation at the end of the game; Tot = total score; You = money taken for self while playing the AlAn's game.

Table 3 also presents correlations between RC and measures of CU (i.e. score derived from the full ICU scale and from the APSD callous subscale); we highlight results for RC but “You” (i.e. AlAn's game earnings) results are also presented and are similar. For both groups combined, RC significantly and negatively correlated with measures of callous-unemotional traits; subjects with higher CU scores tended to leave less for the Red Cross. Similarly, within the control group, RC significantly and negatively correlated with measures of CU; within patients the RC-CU relationship was also negative, albeit smaller in magnitude and nonsignificant.

To further explore and confirm these results, we conducted multiple regression analyses to test whether the relationship between AlAn's game and ICU total score was explained by important covariates (n = 39 subjects); The relationship between ICU total score (dependent variable) and percent of “Yes” answers on Active Trials of the AlAn's game (independent variable) remained significant (p = 0.02) while controlling for group (patient vs. control), externalizing and internalizing problem scales from the Youth Self Report, socioeconomic status, sex, age and race (white vs. other) and ethnicity (Hispanic yes vs. no).

### AlAn's Game Performance

The AlAn's game contained two types of Attention-Control Trials: those in which the logical choice was to accept (gain to the subject, no loss to the Red Cross) and those in which the logical choice was to reject (loss to both the subject and the Red Cross). Patients and controls accepted about 97.5% and 96.5% of the “logically-accept” trials (p = 0.53), respectively, and 0.8% and 0.9% (p = 0.99) of the “logically-reject” trials, respectively (similar results for patients with CU and controls without CU, see Figure 3.D and 3.E; also see Figure S1). This supports that patients and controls both attended to trial content and generally acted logically when non-ambiguous trials were presented. For Calculation Trials, in which subjects were asked “Is the YOU number bigger?” patients and controls performed equally well (patient mean 57.2±3.68 correct out of 60 Calculation Trials; control mean 57.7±3.18; p = 0.61) supporting that patients and controls were equally able to recognize the relative numerical values in the time allowed. Again similar results were seen for patients with CU (56.6) and controls without CU (58.1; p = 0.40).

## Discussion

Our between-group and dimensional analyses support that AlAn's game outcomes are associated with severity of CU traits. However, AlAn's game outcomes did not correlate significantly with severity of conduct problems, nor did these outcomes discriminate patients with CP and controls without CP.

Examination of Figure 3.F suggests that for the AlAn's game, when reward to self is high and deductions from the charity donation are low, group differences are negligible; generally subjects in all groups often accepted them. However, differences between groups are obvious for trials with large deductions from the Red Cross donation or when “You” values were low. We see this effect in our ANOVA analyses, where differences between groups were more obvious with more-costly-to-Red-Cross and low-benefit-to-You trials. We speculate that expanding the matrix to include more severe losses from the Red Cross donation, or including more trials from the upper right quadrant, might strengthen group discrimination.

We carefully designed AlAn's game to control for several factors that could contribute to between-group differences while having little to do with self-benefiting or antisocial tendencies. First, groups might differ in their valuation of the “other”. We hypothesized that our “other”, the Red Cross, would generally be considered beneficent and similar responses on a visual analogue rating across groups confirmed this was the case. Second, groups might have differed in their attention to the game or in their ability to understand relative values in the time allowed; however, we have shown the groups did not significantly differ in Attention-Control or Calculation Trial performance. Third, given that patients differ from controls in decisions requiring assessment of risk [47], patients might have engaged in highly self-benefiting behaviors because they thought harm to others was unlikely. Thus, by design, we carefully told subjects what outcomes would occur. Fourth, adolescent patients with conduct and substance problems may act impulsively [48]. Thus, we required subjects to wait 5 seconds before responding to trials. Other work among antisocial adults has suggested that instituting such a delay normalizes such subjects' reversal learning [49]. Fifth, we required abstinence from drugs and alcohol, thereby excluding very recent drug use as a contributor to group differences. Finally, we presented subjects with the same series of choices (same opportunity) and minimized extraneous stimulation in a private, quiet environment, while using a novel situation, devoid of peer influences.

### Understanding the link between AlAn's and CU

Our results raise the question of why AlAn's game outcomes are related to the construct of CU traits. We posit that AlAn's game captures information about CU traits because it requires decision-making in a socially relevant context where subjects must weigh competing interests: benefit to self vs. harm to another. Work from multiple groups suggests that youth with CP and CU have difficulty reading the emotional states of others (e.g. deficits in processing displays of fear) [50], [51], demonstrate less emotional empathy, positive affect [8], and less empathic concern, are less able to take another's perspective [32] and exhibit deficits in emotional processing [12] and moral reasoning [13]. Some suggest that youth with CP and CU, because of these emotional deficits, are less able to learn from others' responses to their actions, impacting conscience development [14]. Perhaps because of this poor emotional responsivity and diminished autonomic reactivity to provocation [9] such youth are less likely to constrain behaviors which cause distress to others [13]. In the AlAn's game subjects must decide whether to constrain self-benefiting behaviors because of potential cost to a beneficent other. Thus, engagement of moral beliefs and emotional processing is likely part of decision-making in AlAn's game.

Although we wondered whether some patients would be motivated toward other-harmful behaviors, our results do not support this on average for patients or patients with CU. First, if that were the case, we would have expected strong patient-control differences in the “logically-reject” Attention-Control Trials (i.e. “You” lose 2 cents and “RC” loses 32 cents). Acceptance rates on such Attention-Control Trials were quite low for all groups. In addition, if one examines each row of Figure 3 (A, B, D and E), moving from left to right where the loss to RC increases there is a quite systematic decline of percent of “Yes” responses to Active Trials. If patients were motivated to harm others, we would expect the opposite; in other words, while holding benefit-to-self constant and increasing harm to others, those motivated toward other-harm would be more likely, not less likely, to accept. We also wondered, given the literature on empathy and conscience development, whether patients would have only been motivated by self-benefit and disregarded or been unmotivated by other-harm. Examination of Figures 3.A and 3.D again show that while holding self benefit constant, increasing levels of cost-to-others reduced the likelihood of accepting a trial.

Instead, we would conceptualize our results as supporting that patients and controls go through similar deliberations, but that patients, compared to controls, either more highly value a given benefit to self, undervalue a given harm to others, or both. Unfortunately, although the interactions in our ANOVA analyses attempt to address this question, our design does not allow us to absolutely disentangle this concern.

### Limitations

Our results must be interpreted within the context of the study limitations. First, the study sample is modest in size. However, even with modest samples we demonstrate a significant relationship between AlAn's game outcomes and CU trait severity. Second, tests of discrimination were conducted with a clinic-referred sample and a selected control sample. Results must be extrapolated with great caution to youths with CP and CU in community samples. Our analyses, especially correlation analyses, require replication in unselected samples. Third, the sample is cross-sectional and we cannot determine whether AlAn's game performance has any predictive validity for future offending, violence or aggression. Fourth, we utilized only self-report measures of CU. However, such an approach is widely used in the research literature [11]; self report is stable across time and is moderately correlated with parent reports [52]. Use of self-reports is also in some ways advantageous because antisocial attitudes and behaviors may not be as evident to observers, parental reports may be difficult to obtain from the most dysfunctional families and validity of self-report increases from childhood through adolescence, while validity of parent/teacher reports decrease [52]. Fifth, the amounts of money during each choice in the AlAn's game are small. Based on our debriefing interviews, these amounts appeared to be motivating for most adolescent subjects and our behavioral data strongly indicate that the adolescents did believe that the Red Cross would lose something of value if they made certain choices. For example, each horizontal row of Figures 3A and 3B shows a quite systematic decline in acceptance rates as the offers involved greater losses to the Red Cross. Sixth, although the AlAn's game development benefited from previous work on altruism, we did not include any trials in the game with a loss to the subject and gain to the charity; instead our interests squarely focused on whether or not subjects pursued possible self-benefiting behaviors despite consequences to others. Seventh, in our between-group analyses, it is impossible to rule out a relationship between CP and AlAn's game outcomes because many of our adolescent patients with CP also had CU. Although our dimensional analyses help in this regard, future studies might recruit and compare more balanced groups based on presence and absence of CP and CU. Eighth, because of the large number of statistical tests run, our results must be interpreted with some caution; however, our analyses addressed two main questions, whether AlAn's game was associated with CP and separately, CU. The consistency of our results strengthens our confidence in the study findings. Ninth, our correlations across-group must be interpreted cautiously as group differences in callousness might inflate or artificially create significant correlations; however, our within-group correlations are consistent with those results, showing similar direction and magnitude of association, increasing confidence in results of our dimensional analyses. Finally, while it is possible that subjects' behavior while playing the AlAn's game influenced their self report on measures of callous unemotional traits, the relatively high stability of these scores across childhood and adolescence [11] suggests that scores are not markedly influenced by very recent behaviors or experiences. However, future studies might include parent reports of youths' CU traits, or more remotely completed self-reports, to address this important issue.

### Conclusions and future directions

In this preliminary study of the AlAn's game, adolescents with conduct problems and CU traits, compared with controls without CP/CU, were disposed to benefit themselves while harming others even in a novel situation, devoid of peer influences, where anonymity is assured, reciprocity or retribution are impossible, intoxication is absent, when the “other” to be harmed is considered beneficent, and when opportunity is equal across all individuals. AlAn's game scores were associated with measures of CU and therefore may provide an objective measure of CU. Future studies using larger samples with groups balanced on CP and CU are needed to precisely characterize and confirm the associations between the AlAn's game results and CU and other aspects of externalizing behavior. The AlAn's game might be modified in various ways to extend work presented here; for example, future work might (1) include trials with a higher magnitude of costs-to-others, (2) include a second player as the other, rather than a charity or (3) simultaneously assess tendency toward altruistic behaviors (giving up something to benefit others) and behaviors that provide self-benefit while costing others. Future studies may also utilize the AlAn's game or similar paradigms to study the neural activation patterns during self-other considerations.

## Supporting Information

Figure S1 **Percentage of “Yes” answers to trials by group.** Matrix with “You Gain” values (Y axis) and “Red Cross Loses” values (X axis) in cents. Each cell in the matrix represents a single offer. The first column and top row of the matrix are Attention-Control Trials (see Methods). For each cell, each subject's percent of “Yes” responses was calculated across the 2 sessions. Those values were entered into a grand mean for each group (i.e. Patients and separately, Controls) for each cell. Figure S1.A, S1.B. S1.C and S1.D correspond to manuscript Figures 3.A, 3.B, 3.D and 3.E, respectively.
(DOC)

Materials S1 This file contains the AlAn's Game instructions.
(DOC)
