# Supplementary material for: A Behavioral Test of Accepting Benefits that Cost Others: Associations with Conduct Problems and Callous-Unemotionality
Source: PLoS One. 2012 Apr 27;7(4):e36158. doi: 10.1371/journal.pone.0036158 (PMC3338604; doi:10.1371/journal.pone.0036158)
Supplement: Figure S1 — Percentage of “Yes” answers to trials by group. Matrix with “You Gain” values (Y axis) and “Red Cross Loses” values (X axis) in cents. Each cell in the matrix represents a single offer. The first column and top row of the matrix are Attention-Control Trials (see Methods). For each cell, each subject's percent of “Yes” responses was calculated across the 2 sessions. Those values were entered into a grand mean for each group (i.e. Patients and separately, Controls) for each cell. Figure S1.A, S1.B. S1.C and S1.D correspond to manuscript Figures 3.A, 3.B, 3.D and 3.E, respectively. (DOC) [file pone.0036158.s001.doc]

**S1.A Patients (n=20)**

YOU GAIN (cents)

| **-2** |  | 0 |  | 0 |  | 2.5 |  |
| --- | --- | --- | --- | --- | --- | --- | --- |
| **+2** | 95 | 70 | 80 | 50 | 45 | 40 | 50 |
| **+4** |  | 82.5 | 70 | 52.5 | 67.5 | 47.5 | 45 |
| **+8** | 97.5 | 95 | 80 | 67.5 | 62.5 | 42.5 | 47.5 |
| **+16** |  | 95 | 92.5 | 82.5 | 82.5 | 57.5 | 62.5 |
| **+32** | 100 | 97.5 | 95 | 100 | 87.5 | 77.5 | 65 |
| **+64** |  | 95 | 97.5 | 95 | 95 | 95 | 82.5 |
| YOU GAIN (cents) | **0**  RED CROSS LOSES (cents) | **-2** | **-4** | **-8** | **-16** | **-32** | **-64** |

**S1.B** **Controls (n=19)**

**S1.D** **Control-NoCU (n=16)**

| **-2** |  | 2.6 |  | 0 |  | 0 |  |
| --- | --- | --- | --- | --- | --- | --- | --- |
| **+2** | 94.7 | 71.1 | 55.3 | 42.1 | 23.7 | 23.7 | 26.3 |
| **+4** |  | 78.9 | 65.8 | 34.2 | 50 | 28.9 | 26.3 |
| **+8** | 97.4 | 94.7 | 78.9 | 65.8 | 50 | 31.6 | 31.6 |
| **+16** |  | 97.4 | 94.7 | 84.2 | 65.8 | 47.4 | 47.4 |
| **+32** | 97.4 | 94.7 | 97.4 | 94.7 | 71.1 | 68.4 | 44.7 |
| **+64** |  | 94.7 | 94.7 | 92.1 | 92.1 | 78.9 | 65.8 |
|  | **0** | **-2** | **-4** | **-8** | **-16** | **-32** | **-64** |

RED CROSS LOSES (cents)

**S1.C** **Patients-CU (n=14)**

| **-2** |  | 0 |  | 0 |  | 3.6 |  |
| --- | --- | --- | --- | --- | --- | --- | --- |
| **+2** | 92.9 | 78.6 | 89.3 | 60.7 | 53.6 | 53.6 | 57.1 |
| **+4** |  | 82.1 | 78.6 | 60.7 | 82.1 | 57.1 | 57.1 |
| **+8** | 100 | 92.9 | 82.1 | 78.6 | 71.4 | 50.0 | 57.1 |
| **+16** |  | 92.9 | 89.3 | 85.7 | 82.1 | 67.9 | 67.9 |
| **+32** | 100 | 96.4 | 92.9 | 100 | 89.3 | 82.1 | 75.0 |
| **+64** |  | 92.9 | 96.4 | 92.9 | 92.9 | 92.9 | 85.7 |
|  | **0**  RED CROSS LOSES (cents) | **-2** | **-4** | **-8** | **-16** | **-32** | **-64** |

| **-2** |  | 3.1 |  | 0 |  | 0 |  |
| --- | --- | --- | --- | --- | --- | --- | --- |
| **+2** | 93.8 | 65.6 | 46.9 | 31.3 | 9.4 | 9.4 | 12.5 |
| **+4** |  | 75.0 | 59.4 | 21.9 | 40.6 | 15.6 | 12.5 |
| **+8** | 96.9 | 93.8 | 75.0 | 59.4 | 40.6 | 18.8 | 18.8 |
| **+16** |  | 96.9 | 93.8 | 81.3 | 59.4 | 37.5 | 37.5 |
| **+32** | 96.9 | 93.8 | 96.9 | 93.8 | 65.6 | 62.5 | 34.4 |
| **+64** |  | 93.8 | 93.8 | 90.6 | 90.6 | 75.0 | 59.4 |
|  | **0** | **-2** | **-4** | **-8** | **-16** | **-32** | **-64** |

RED CROSS LOSES (cents)
