# Supplementary material for: A Behavioral Test of Accepting Benefits that Cost Others: Associations with Conduct Problems and Callous-Unemotionality
Source: PLoS One. 2012 Apr 27;7(4):e36158. doi: 10.1371/journal.pone.0036158 (PMC3338604; doi:10.1371/journal.pone.0036158)
Supplement: Materials S1 — This file contains the AlAn's Game instructions. (DOC) [file pone.0036158.s002.doc]

# Alan’s (Altruism/Antisocial) Game Practice Instructions

**PRACTICE SESSION ON LAPTOP COMPUTER:**

- “Welcome. You are going to play Alan’s Game. It’s a game where you have the right to earn money for making choices. You will get to decide how much you earn.
- You just watched a movie about good things that the Red Cross does. The researchers are going to give up to $16 to help the Red Cross do its work. But when you make *more* money in Alan’s Game, the Red Cross will get *less* money. It’s up to you to decide how much you should get, and how much the Red Cross should get.
- There are no right or wrong answers – it’s just up to you. And the researchers don’t plan to tell your parents how much money the Red Cross got. If you’re a patient we won’t tell your treatment program either.
- During the game you will get to make choices like this one. During the game, you will make lots of these choices, one after another.

- Each time a choice appears on the screen, you will push buttons with either your left finger or your right finger.
- This is your left index finger.
- This is your right index finger.
- You either press this YES button with your left finger
- or this NO button with your right finger. Don’t push any buttons yet – wait until I tell you to press the buttons for practice.
- Here are the thermometers. They will show up after you make a choice. They tell you how much money you have earned and how much we will donate to the Red Cross. You start with no money. The Red Cross starts with 16 dollars.
- Put your fingers on the buttons now. The researchers will help you if you need it. Don’t press any buttons until I tell you to.
- When the game begins, you will see a choice like this one. Your choice is always just YES or NO. At the top there is a question, “Change both thermometers?”
- Below it says “You, plus-thirty-two cents”
- and “Red Cross, minus-eight cents”. What you will get will always be on the left. What will happen to the Red Cross donation will always be on the right.
- Pressing with your left finger will always mean “Yes” and pressing with your right finger will always mean “No”. If you want to go along with a choice, you push the left YES button. Then both thermometers will make the change that is shown. If you don’t want to go along with a choice, push the right NO button. Then neither thermometer will change that time.
- The game will give you a few seconds to think about each choice and to decide “Yes” or “No”.
- At the bottom of the screen there is a circle. When that circle turns from red to green, that’s when you push the button. In the game when the circle turns green, you must push the “YES” button OR the “NO” button REALLY FAST.
- Pressing the NO button means “No, don’t change the thermometers this time.” If you press the NO button, neither counter will change. Pressing the YES button means “Yes, change the thermometers this time.” In this choice if you press the YES button, the thermometers will change, you will get thirty two cents, and the Red Cross donation will go down by eight cents.
- Just for practice now, let’s make a choice.
- This time let’s choose “Yes” – press the YES button
- Then the thermometers will appear. Remember, you started with no money, so your thermometer started at zero.
- The Red Cross donation started at 16 dollars. So the Red Cross thermometer was at 16.
- Since you said you wanted to “Change the Thermometers” the thermometers have changed. This time you added thirty two cents and the Red Cross donation went down by eight cents.
- Before the next choice you will see a blank screen with a plus sign. At those times just look at the screen.
- During the game you will always choose either YES or NO. You decide. There is no wrong answer. The Red Cross will end up with some money. You should end up with some money too.
- Remember, when the circle turns green, press REALLY FAST. If you wait too long to make a choice, the computer will just skip that one and you will not get any money for that choice.
- Now, there is a catch, so stay awake. Sometimes in the game you can lose money. Here is a choice that shows that. The left box shows “You, minus 2 cents”; that means you will lose 2 cents if you press “Yes”. The other box says that the Red Cross will lose four cents if you press “Yes”. You decide whether you want to change the counters. If you press NO on this one you will not lose any money and the Red Cross will not lose any money. If you press YES on this one, your thermometer will go down by two cents and the Red Cross thermometer will go down by four cents.
- In the game there are also screens like this one. At the top of the screen it says “Is the ‘You’ number bigger?” instead of “Change both thermometers?”
- Below it says “You plus 4 cents”
- and “Red Cross plus 8 cents”. You need to decide whether the “You” number is bigger than the “Red Cross” number. If the “You” number is bigger than the “Red Cross” number, then press “Yes”. If the “You” number is not bigger than the “Red Cross” number then press “No”. This time the “You” number is 4 and the “Red Cross” number is 8. The “You” number is not bigger, so you should press “No”.
- At the bottom of the screen there is a circle. When that circle turns from red to green, that’s when you push the button. In the game when the circle turns green, you must push the “YES” button OR the “NO” button REALLY FAST.
- Then the thermometers will appear. For these screens that ask “Is the You number bigger?” the thermometers won’t change. But at the end of the game, if you have done a good job of deciding whether the “You” number was bigger than the “Red Cross” number, you will get 25 extra cents.
- You will make lots and lots of choices during the game. Although the amounts of money are small for each choice, there are many choices, Depending on your choices, you can earn between about 1 cent and $16. The Red Cross can end up with anywhere from 4 cents to $16.
- At the end of the game, we will check the thermometers. At the end of the game, we will pay you whatever you have earned. The Red Cross’ thermometer is here. After the game, we will donate the money in the Red Cross thermometer to the Red Cross.
- Do you have any questions?
- Ok, then let’s practice the game. You will get to make choices to get used to the game. This is only practice and does not count.

FINAL INSTRUCTIONS:

- Now we’re going to have you play the game for real. It’ll take about 30 minutes. You’ll play for about 15 minutes, have a very short break, where a picture of the ocean will appear. And then you will play for 15 more minutes. After you begin don’t ask any questions. If you stop, you can’t start again. Remember that all choices are real. You really get the money from your choices. The Red Cross will really get a donation.
